# Adipose Tissue-Specialized Immunologic Features Might Be the Potential Therapeutic Target of Prospective Medicines for Obesity

**DOI:** 10.1155/2017/4504612

**Published:** 2017-03-30

**Authors:** Fan Yao, Ming Zhang, Li Chen

**Affiliations:** ^1^Department of Pharmacology, College of Basic Medical Sciences, Jilin University, Changchun 130021, China; ^2^School of Nursing, Jilin University, Changchun 130021, China

## Abstract

Excessive lipid accumulation in adipose tissue is either the source of obesity or the cause and result of chronic local inflammation, and recent studies indicate that the accumulation may induce many other specialized immunologic features with macrophages and epidemic diseases. We analyze the effective stages of immune cells in adipose tissue, including macrophage recruitment, macrophage polarization, and macrophage-like phenotype preadipocyte possession to find optimal sites as drug targets. Subsequently, some main signaling pathways are summarized in this review, including the AMP-activated protein kinase (AMPK) pathway, the JNK signaling pathway, and a novel one, the Notch signaling pathway. We illustrate all these points in order to determine the general pathogenesis of chronic low-grade local inflammation in adipose tissue and the related signaling pathways. In addition, signal-associated prospective compounds, such as berberine, are summarized and discussed with potential targets in pathogenesis. This might provide some possible thoughts and novel therapies for studying chronic inflammatory diseases, such as insulin resistance and type 2 diabetes mellitus.

## 1. Introduction

The World Health Organization (WHO) reported that more than 1.4 billion adults and older individuals were overweight in 2012. More than 200 million men and approximately 300 million women were classified as obese. Additionally, more than 40 million children under the age of 5 were classified as being obese in 2012. As a worldwide and dangerous epidemic, obesity increases the risk of some chronic diseases and induces low-grade inflammation with many other complications, such as atherosclerosis, cardiovascular problems, insulin resistance, and type 2 diabetes mellitus [1–5]. Adipose tissue also plays a crucial role in the generation of low-grade inflammation and progression. In hypertrophic adipose tissue, many cytokines and chemokines are important contributors in different regulatory pathways, especially cytokines that are activated and released by macrophages/monocytes immune cells. In this review, we will summarize the relevant reports and research about chronic low-grade inflammation in adipose tissue to find prospective medicines. The process contains the chronic local inflammation mechanism of immune cells in adipose tissue, such as macrophage recruitment, macrophage polarization, and macrophage-like phenotype preadipocyte. Subsequently, pertinent signaling pathways will be illustrated and generalized involving the adenosine monophosphate-activated protein kinase (AMPK) signaling pathway and the Notch pathway for potential medical targets.

## 2. Origin of Macrophages/Monocytes in Chronic Inflammation of Local Adipose Tissue

In the foetal stage, the yolk sac and foetal liver haematopoiesis are the embryonic precursors for macrophages [6, 7]. Most of the tissue-resident macrophages were thought to arise from embryonic precursors [8, 9]. However, a novel method has helped illuminate the fact that the sole origin of tissue-resident macrophages in the liver and pancreas might be yolk sac macrophages. This leads to the origin of tissue-resident macrophages being a controversial topic [7, 10]. In the postnatal stage, blood monocytes developed from haematopoietic stem cells in the bone marrow are the main precursors of macrophages in the circulation system [11–13]. Similarly, T cells promote monocyte differentiation and macrophage polarization [14]. These should be the classical approach for macrophage production. However, currently, researchers have found that macrophages/monocytes have been included in the adipose tissue for regulation of chronic local inflammation. Although the mechanism of chronic inflammation has not been clearly elucidated, the possible modulating methods and pathways have been reported and discussed. The adipose tissue contains adipocytes, preadipocytes, stem cells, and other stromal cells; macrophages as immune cells and homeostatic regulators make up almost 10% of the normal adipose tissue. Macrophages from normal adipose tissue take the function of anti-inflammation to maintain homeostasis. In contrast, when the homeostasis is broken up, as in obesity, the number of macrophages will increase significantly, contributing up to 50% of the cells in adipose tissue. These cells contain 85% of macrophages from recruitment and infiltration as well as 15% from preadipocyte macrophage-like phenotypes [15–17]. Any proposed medicine should play a key role in modulating the large amount of macrophages in adipose tissue for disease treatments.

### 2.1. Macrophage Recruitment and Infiltration

Under diet-induced obesity conditions, the activation of macrophages results in chronic low-grade inflammation in local adipose tissue. This could be induced by macrophage recruitment. Lumeng et al. [18] reported numerous F4/80^+^ CD16/32^+^ CD11c^+^ macrophages accumulating in visceral adipose tissue. Another important difference is the higher expression of proinflammatory factors such as tumour necrosis factor alpha (TNF-*α*), interleukin-1*β* (IL-1*β*), and inducible nitric oxide synthase (iNOS). The exact mechanism remains a topic of study. It is already understood that the activated monocytes appear to accumulate in local tissue that is stimulated via cytokines such as MCP-1 secreted by macrophages. The recruitment and infiltration of monocytes/macrophages might be associated with adipocyte hypertrophy, local hypoxia, and the interaction between adipocytes and macrophages [19]. Moreover, MCP-1 is a type of inflammation-related genetic code cytokines that plays a critical role in the pathways of macrophage recruitment and the regulation of insulin sensitivity. Previous studies of obese processes illustrated that increased adipocyte volume could induce angiogenesis and increase adipose tissue consumption. When the rate of increased adipocyte volume is not equal to the rate of vascular proliferation, lipids excessively accumulate in adipocytes. Subsequently, anoxia tissue leads to a volume increase in adipocytes as well as cell apoptosis. Then, excessive amounts of saturated fatty acid and accumulated cytokines are released [20]. In detail, the regulating pathways might be highly associated with the proteins peroxisome proliferator-activated receptor *γ* (PPAR*γ*), nuclear factor-*κ*B (NF-*κ*B), and MCP-1.

### 2.2. Macrophage Polarization

The macrophages in chronic inflammation could also come from macrophage polarization. This could be regulated by many factors. Based on the phenotype and a variety of secreted cytokines, there are two types of macrophages, classically activated M1 and alternatively activated M2. It is important to note that M1 has the function of proinflammation by secreting cytokines such as TNF-*α*, IL-1*β*, iNOS, and MCP-1 [21]. M2 takes on the function of anti-inflammation and tissue regeneration. With the help of IL-4, Arg-1, IL-10, and TGF-*β*, macrophages might be induced to polarize M2 and secrete cytokines such as TGF-*β*, VEGF, and EGF or additional Arg-1 and IL-10. In the final stage of inflammation, M2 enhances tissue repair and fibrosis [22, 23]. Additionally, two crucial nuclear factors, PPAR*γ* and NF-*κ*B, are also the key targets in the modulation of macrophage polarization. Furthermore, PPAR*γ* has been proven to be the important factor of M2 generation. It activates M2 polarization with the function of anti-inflammation. If PPAR*γ* has not been activated, the alternatively activated pathway will be blocked [24, 25]. Meanwhile, PPAR*γ* blocked the proinflammatory pathway of NF-*κ*B and inhibited the expression of relative factors such as TNF-*α* [26]. Therefore, macrophage polarization is one of the important processes in chronic inflammation. Moreover, in a diet-induced obese mouse model, macrophage polarization can be regulated for influencing obesity-induced adipose tissue inflammation or insulin resistance. This might occur in interferon tau therapy, in chronic *Trypanosoma cruzi* infection, or with miR-130b assistance. This procession is also correlated with PPAR*γ* [27–29]. Therefore, in local adipose tissue, macrophage polarization plays an important role in local chronic inflammation and insulin resistance.

### 2.3. Macrophage-Like Phenotype Preadipocytes

Macrophage-like phenotype preadipocytes are thought to originate from undifferentiated white adipocytes. The lineage tracing of adipose progenitors are generalized first. Adipocytes contain white adipocytes, brown adipocytes, and beige adipocytes that resemble white adipocytes but have the same function of classical brown adipocytes [30]. All of them originate from mesenchymal precursor cells. The majority of white adipocytes derive from Myf5^−^ precursors and some white adipocyte precursors originate from the Myf5^+^ lineage. Mature white adipocytes derive from white adipocyte precursors or named preadipocytes in adulthood [31–34].

Based on previous studies, a question has developed about the relationship between adipocytes and macrophages. First, the major mediators of inflammation, for example, ILs and TNF-*α*, have been found to be secreted from adipocytes and macrophages. Additionally, leptin takes part in not only proinflammation but also in energy metabolism, in particular, through T lymphocyte proliferation and macrophage activity [35]. In the recent years, the new definition of cell reprogramming has become an increasingly popular concept in advanced fields. Except for macrophage recruitment and polarization, other immune cells for local inflammation of adipose tissue may come from macrophage-like phenotype preadipocytes; we are working to identify these macrophage-like type preadipocytes and explore their functions. This kind of possible reprogramming means not only stem cell proliferation and differentiation but also genetic expression reprogramming that is induced by stimuli and changes in cell phenotype or function under certain conditions [36]. Adipose tissue is the potential area to generate cell reprogramming. For preadipocytes, they could be differentiated from adipose stem cells. Under chronic inflammation conditions, preadipocytes result in local inflammation caused by cytokines and might appear as macrophage phenotypes. The preadipocytes are stopped from differentiating into adipocytes [37]. Moreover, some findings confirm that there is an essential nuclear factor, PPAR*γ*, that mediates the process of preadipocytes differentiating into adipocytes.

## 3. Related Signaling Pathways

In addition to immune cells studies in chronic local inflammation mechanism, several related signaling pathways have been found and explored. These pathways include the adenosine monophosphate-activated protein kinase (AMPK) signaling pathway, the C-Jun N-terminal kinase (JNK) signaling pathway, the Notch signaling pathway, the PI3K/Akt signaling pathway, and the JAK/STAT signaling pathway, which have been studied in the last few decades. The first three signaling pathways will be generalized below; they are linked with the chronic low-grade inflammation mechanism. In addition, the key signals might become the targets of prospective drugs in further studies.

### 3.1. Adenosine Monophosphate-Activated Protein Kinase (AMPK) Signaling Pathway

AMPK is a critical regulator in energy metabolism at the molecular level, in particular in glucose metabolism [38–40]. AMPK in the hypothalamus is a common regulator of weight gain. As a stress sensor, AMPK can be activated by a cluster of factors such as oxidative, metabolic, and physical stresses [39, 41, 42]. According to the heterotrimeric complex with one catalytic *α* subunit and two regulatory units *β* and *γ*, AMPK contains two isoforms of *α* (*α*1 and *α*2) and *β* (*β*1 and *β*2) with three *γ* subunits (*γ*1, *γ*2, and *γ*3). The distinctive structure of AMPK isoforms is expressed differently in mammalian tissues [43]. Furthermore, AMPK is a key modulator in type 2 diabetes that is related to insulin sensitivity. This may also be mainly associated with obesity-induced inflammation and insulin sensitivity. Additionally, in adipocytes, the accumulation of lipids, together with the ectopic storage of fat in the pancreas, muscles, and other internal organs, may stimulate immune system defense and could provoke proinflammatory cytokine secretion and macrophages/monocytes recruitment, especially in adipose tissue [43–45]. With the help of several pharmacological activators or adipokines, AMPK inhibited the inflammatory reaction. For instance, the inflammatory response induced by lipopolysaccharide (LPS) was inhibited by activating AMPK with 5-aminoimidazole-4-carboxamide riboside (AICAR), a classical activator of AMPK [43, 46]. Another example for consideration is the findings of Steinberg et al. [47] who demonstrated that tumour necrosis factor-*α* (TNF-*α*) might suppress AMPK activity by aggravating the expression of protein phosphatase 2C, an inhibitor of the AMPK signaling pathway.

**Figure 1:**
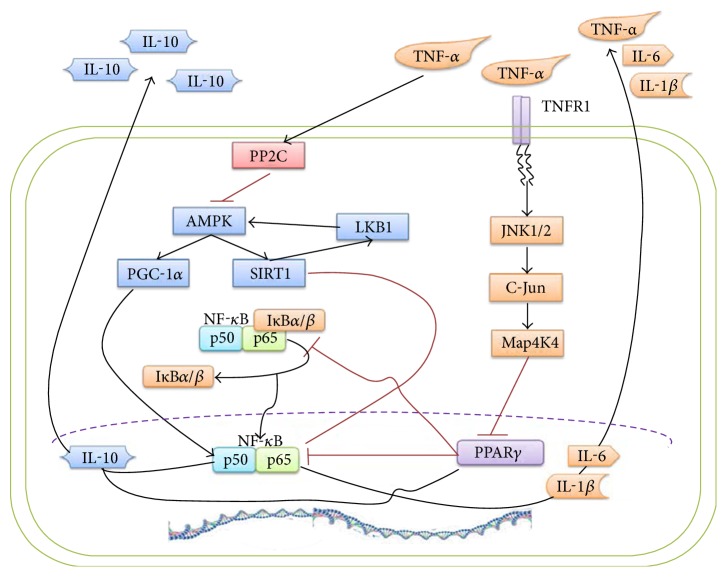
The AMPK and JNK signaling pathways are shown above. Besides the circulatory regulation of AMPK-SIRT1-LKB1, AMPK indirectly regulates NF-*κ*B p65 by SIRT1. Additionally, AMPK might induce the association of PGC-1*α*. The interaction of NF-*κ*B and I*κ*B might result in nuclear accumulation and activation of NF-*κ*B, leading to IL-10, IL-1*β*, and IL-6 transcriptions and expressions. Likewise, TNF-*α* inhibits AMPK via upregulation of protein phosphatase 2C (PP2C). In the JNK signaling pathway, the main pathway is that TNF-*α* activates Map4k4 by combining with TNFR1 to inhibit PPAR*γ*. Nevertheless, TNF-*α* activates NF-*κ*B by phosphorylation of JNK1/2. PPAR*γ* might block the interaction of NF-*κ*B and I*κ*B.

In the AMPK signaling pathway, AMPK inhibited NF-*κ*B p65 phosphorylation, suppressed gene expression of proinflammatory adipocytokines, and upregulated PPAR*γ* expression [48]. Specifically, AMPK indirectly inhibited NF-*κ*B signaling through some downstream mediators, such as silent information regulator 1 (SIRT1) and peroxisome proliferator-activated receptor *γ* coactivator 1*α* (PGC-1*α*) [43]. In the AMPK-SIRT1-NF-*κ*B signaling pathway, there is a feedback loop during the energy deficiency. SIRT1 deacetylase was activated by AMPK via increasing cellular NAD^+^ levels. In contrast, AMPK was activated by LKB1 activity that is stimulated by SIRT1 [49, 50]. Additionally, the acetylation of NF-*κ*B p65 improved its transactivation capacity. Conversely, SIRT1 interacted with p65 and finally deacetylated the p65 protein at lysine 310. Thus, AMPK inhibited NF-*κ*B signaling via SIRT1-induced deacetylation [43, 51]. Similarly, fatty acids are involved in the AMPK-PGC-1*α*/NF-*κ*B signaling pathway, inducing the association of PGC-1*α* factor with NF-*κ*B p50 in hepatocytes. Moreover, PGC-1*α* and p50 might bind to the IL-10 promoter and lead to the expression of IL-10 cytokine [52], because NF-*κ*B is a key mediator in fat-induced inflammation. Both of these indirect regulations in adipocytes by AMPK demonstrated that the AMPK signaling pathways could be crucial mediation pathways in adipose tissue chronic inflammation (Figure 1).

### 3.2. C-Jun N-Terminal Kinase (JNK) Signaling Pathway

The JNK signaling pathway belongs to the superfamily of mitogen-activated protein kinases (MAPKs) and is a major modulator of cell proliferation, differentiation and apoptosis [53–55]. There are three different isoforms, JNK1, JNK2, and JNK3, in the JNKs family; however, only the Jnk1 and Jnk2 genes can be expressed ubiquitously in all tissues [56, 57]. Related JNK proteins are expressed ubiquitously in most tissues [55]. Among these tissues, JNK in macrophages plays a significant role in the establishment of fat-induced insulin resistance and chronic inflammation, as well as macrophages accumulation and proinflammatory macrophage polarization [18, 58].

Furthermore, in the JNK signaling pathway, JNK1/2 was one of the indirect mediators for the process in which TNF-*α* decreased PPAR*γ* and glucose transporter isoform 4 (GLUT4) expressions in adipocytes [59]. Namely, TNF-*α* maintained prolonged activation of JNK1/2 via TNF-*α* receptor 1 (TNFR1) and phosphorylates JNK downstream transcription factor c-Jun that restrained Map4k4 mRNA expression. In addition, Map4k4 expression aggravated the inhibition of PPAR*γ*, while TNF-*α* activated NF-*κ*B by phosphorylation of JNK1/2 and p38 [59]. These two modulators are associated with adipose tissue inflammation and insulin resistance. Therefore, the JNK pathway could also be a useful signaling pathway in the treatment of obesity-induced syndromes and diseases (Figure 1).

### 3.3. Notch Signaling Pathway

The Notch signaling pathway could maintain tissue renewal by promoting or inhibiting cell proliferation, cell differentiation, and cell death [60]. The Notch family has four transmembrane Notch receptors expressed in mammals that may combine with the Notch signal and regulate distinct downstream factors [55]. Some experiments have shown that pharmacological suppression of Notch signaling in obese mice augmented Ucp1 expression, reduced blood glucose, and ameliorated obesity [61].

**Figure 2:**
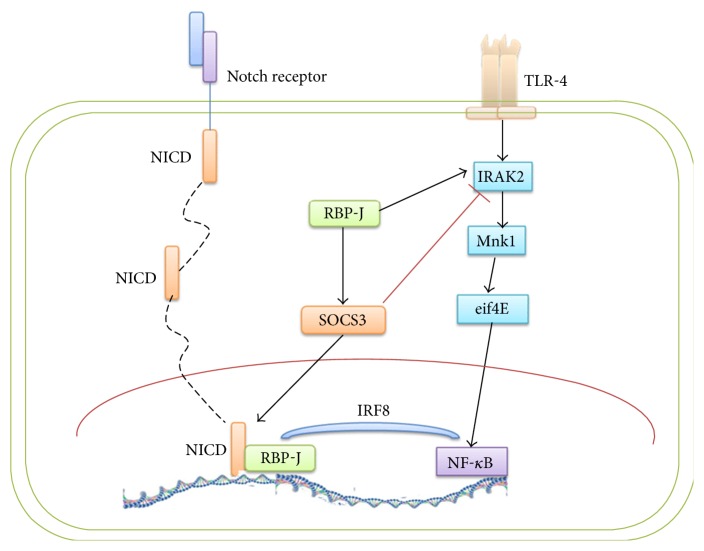
The Notch signaling pathways are shown mainly above. The Notch receptor on the cell membrane combines with a DII4 ligand and induces the translocation of NICD from cytoplasm into cell nucleus, binding with RNP-J. Likewise, TLR4 could play a role in Notch signaling pathway to regulate IRAK2, eif4E, and NF-*κ*B, activating the RBP-J signal. However, SOCS3 inhibits one key protein IRAK2 among the signals above.

In addition to the function of glucose reduction and obesity alleviation, the Notch signal modulated macrophage polarization. The activation of A disinterring and metalloproteinase (ADAM) domain-type proteinase and *γ*-secretase complex were induced by the combination of the Notch receptor with the ligand, DII4 [62]. The Notch intracellular domain (NICD) translocated into the nucleus and bound with the sequence-specific DNA-binding factor RBP-J [55]. These regulations may lead to M1-like polarization. Additionally, the synthesis of interferon regulatory factor 8 (IRF8), a transcription factor, might be promoted by RBP-J via selectively augmenting IRAK2-Mnk1-eif4E axis signaling of TLR4 [62]. TLR4 can both upregulate NF-*κ*B and activate RBP-J. Conversely, SOCS3 plays a crucial role in inhibition of M1-like polarization in the downstream regulation pathway [55, 63]. The Notch signaling pathway has recently been a focus of research. Thus, the main topic might be whether the Notch could be the mediator and potential treatment strategy in macrophage recruitment or other immune cell interactions in adipose tissue inflammation (Figure 2).

## 4. Prospective Compounds for Local Adipose Tissue Chronic Inflammation and Obesity-Induced Insulin Resistance

Based on the above signaling pathways, prospective compounds have been studied for ameliorating local adipose tissue chronic inflammation with obesity-induced insulin resistance, also known as metaflammation [64]. Bitter melon has been found to ameliorate insulin resistance partly by modulating the inflammatory status. Specifically, it might block NF-*κ*B by degradation of I*κ*B*α* and suppress phosphorylation of JNK/p38 MAPKs, which were observed not only in epididymal fats but also in the liver and muscle [65, 66]. For JNK or MAPK signals, benzenediamine derivative FC98 also reduced insulin resistance against metaflammation based on a model of diet-induced obese C57BL/6J mice with the effect of JNK and p38 [67]. Another potential flavonol, (-)-epicatechin, prevented TNF-*α*-triggered activation of inflammation and insulin resistance via suppressing JNK or ERK phosphorylation [68].

More prospective compounds might also interact with proteins in the AMPK signaling pathway. It has been reported that brown alga *Ecklonia cava* polyphenol extract or quercetin with antioxidative or anti-inflammatory activities modulated AMPK and SIRT1 by reducing adipose tissue mass and lipid accumulation or attenuating macrophage recruitment in epididymis adipose tissue [69, 70]. In detail, quercetin as flavonol-attenuated adipogenesis in 3T3-L1 cells activated the expression of AMPK or decreased the levels of phosphorylated ERK and JNK and inhibited PPAR*γ* and enhancer-binding protein *α* (C/EBP*α*) in mRNA and protein expressions [71, 72]. It was studied in primary bone marrow-derived macrophages or macrophage cell lines (human U937 monocytes, murine J774 macrophages) and human adipocytes to prove that quercetin possessed the functions of anti-inflammation in local adipose tissue. Quercetin decreased the secretion of TNF-*α*, IL-6, and IL-1*β* and increased IL-10 production. Additionally, it inhibited NO production and iNOS expression by blocking NF-*κ*B, the transcription factor of iNOS [73–75]. Therefore, this evidence explains anti-inflammatory activity of flavonoid quercetin. Similarly, berberine, the extract of rhizoma coptidis, has been studied for several years regarding the activation of AMPK with metabolic effects. It has been used and studied for its properties of reducing body weight, plasma triglycerides, and some inflammatory cytokines or chemokines [76, 77]. As a derivative of berberine, nandinine has also been reported to have an activation effect in AMPK and blocking I*κ*B-*β* activation for attenuating insulin resistance in adipocytes [78]. Additionally, berberine strongly suppressed MCP-1 production in macrophages and affected the level of leptin and the expression of PPAR*γ* with downregulation of TNF-*α*, IL-6, and so forth [79, 80]. The alkaloid berberine, as a traditional Chinese medicine, could regulate AMPK directly and suppress adipokine and inflammatory cytokine production by affecting protein expression in the inflammation pathway, which might be a prospective drug for ameliorating obesity-associated chronic inflammation and improving insulin sensitivity. Both the structural analysis and the pharmacological mechanism should be studied further. However, all the regulating approaches above might provide some thoughts and targets for drug development about adipose tissue-specialized immunologic features or relevant diseases.

## 5. Conclusion

Generally, as the number of obese people increases, a variety of obesity-related diseases and low-grade chronic inflammation cause injury in people's lives and health to different extents. These relative complications might obviously influence the health and quality of daily life. For obesity-induced inflammation, the possibility of macrophage accumulation has been concluded to have three aspects: macrophage recruitment or infiltration, macrophage polarization, and macrophage-like phenotype preadipocytes. All these aspects shed light on the functional plasticity of macrophages and provide potential therapies to regulate of obesity-induced chronic inflammation. Moreover, AMPK, JNK, and Notch signaling should be the critical pathways for regulating macrophage and adipose tissue local inflammation. These pathways are associated with PPAR*γ* or NF-*κ*B to some extent. Therefore, the aim of the research on the obesity-induced local chronic inflammation mechanism might be focused on the mediators PPAR*γ* or NF-*κ*B, potentially the key points for regulating inflammation. The relationships between PPAR*γ* and NF-*κ*B or among PPAR*γ*, NF-*κ*B, and other relevant pathways should also be discussed. It could be another interesting debate whether there is a factor or molecule that could inhibit or activate these mediators and classical signaling pathways to become biomarkers in the treatment of local chronic inflammation, insulin resistance, and type 2 diabetes. Because berberine could modulate AMPK for improving insulin sensitivity, how berberine regulates AMPK and downstream proteins for anti-inflammation in local adipose tissue should be elucidated in detail. Similarly, adipose tissue as an immune organ might explain the pathogenesis of local insulin resistance and systemic insulin resistance induced by obesity in a local inflammatory method or low-grade inflammation. These discoveries could provide novel therapies or detection strategies for obesity-related inflammation and complications.
